# Cognitive functions in people with mental disorders: focus on self-reflection, insight and mindwandering

**DOI:** 10.1192/j.eurpsy.2023.1937

**Published:** 2023-07-19

**Authors:** A. Natale, L. Fusar-Poli, S. Sturiale, V. Placenti, M. Marino, A. Amerio, A. Rodolico, C. Concerto, A. Aguglia, A. Petralia, M. S. Signorelli, G. Serafini, M. Amore, E. Aguglia

**Affiliations:** 1 Department of Clinical and Experimental Medicine, University of Catania, Catania; 2 Department of Brain and Behavioral Sciences, University of Pavia, Pavia; 3 Department of Neuroscience- Rehabilitation- Ophthalmology- Genetics- Maternal and Child Health- Section of Psychiatry, University of Genoa; 4 IRCCS Ospedale Policlinico San Martino, Genoa, Italy

## Abstract

**Introduction:**

People with mental disorders may present impairments in cognitive and metacognitive functions. Self-reflection is the ability to reflect on oneself (specifically on one’s behavior, emotions, and thoughts) and insight is the awareness of one’s internal experience. Mindwandering (MW) is defined as the tendency to divert attention from current reality without a clearly defined intention. It can be spontaneous or deliberate. Several studies have investigated these alterations in patients with schizophrenia (SZ), while less is known for people with substance use disorder (SUD).

**Objectives:**

The aim of the present study was to explore self-reflection, insight and MW in a group of patients with SZ and SUD.

**Methods:**

The Self-reflection and Insight Scale (SRIS) and the spontaneous (MW-S) and deliberate (MW-D) mindwandering scales were administered to 25 patients with SZ, 21 patients with SUD, and 21 healthy controls (HC). Linear regressions were performed to evaluate the associations between the variables under study.

**Results:**

Preliminary data showed that SZ and SUD patients presented lower SRIS and MW values than HC. Examining MW in detail, participants with SZ reported higher scores at than MW-D, while in people with SUD, MW-D scores were higher than MW-S scores. Linear regressions revealed that MW-D was negatively associated with self-reflection in SUD; moreover, insight scores were negatively associated with MW-S in SZ.

**Conclusions:**

Our preliminary results confirm the importance of acting on the elements of metacognition in patients with mental disorders to improve the general outcome of the disease. A comprehensive therapeutic approach should include psychotherapeutic and social interventions aimed at increasing attention and introspection.

**Disclosure of Interest:**

None Declared

